# Targeting AMPK for Cancer Therapy: Metabolic Reprogramming as a Therapeutic Strategy

**DOI:** 10.32604/or.2025.067487

**Published:** 2025-09-26

**Authors:** Minseo Hong, Jea-Hyun Baek

**Affiliations:** School of Life Science, Handong Global University, Pohang, 37554, Republic of Korea

**Keywords:** Cancer metabolism, cancer therapy, AMP-activated protein kinase (AMPK), drug repurposing, drug repositioning

## Abstract

AMP-activated protein kinase (AMPK) is a highly conserved serine/threonine kinase that functions as a central regulator of cellular energy status. In cancer, where metabolic reprogramming enables rapid proliferation and survival under stress, AMPK functions as a metabolic checkpoint that restrains tumor growth by inhibiting biosynthetic pathways and promoting catabolic processes, such as autophagy and fatty acid oxidation. Given its role in opposing many hallmarks of cancer metabolism, AMPK has attracted significant interest as a therapeutic target. This review examines the molecular mechanisms by which AMPK influences tumor progression and evaluates the preclinical and clinical evidence for pharmacological AMPK activation using agents such as metformin, phenformin, and canagliflozin. While promising anti-tumor effects have been reported in specific contexts—such as HER2-positive breast cancer, colorectal cancer, and metabolically distinct lung cancer subtypes—clinical efficacy remains variable. Limitations include indirect activation mechanisms, low tissue penetrance, tumor heterogeneity, and lack of reliable biomarkers for patient selection. We discuss emerging strategies to overcome these challenges, including combination therapies, metabolic stratification, and the development of direct AMPK activators or mRNA-based delivery platforms. Together, these insights support a renewed focus on AMPK as a modifiable node in cancer metabolism and a candidate for integration into precision oncology frameworks.

## 1 Introduction

Cancer remains one of the leading causes of death globally, with incidence and mortality rates continuing to rise. According to the World Health Organization (WHO), cancer accounted for nearly 10 million deaths in 2020, and by 2023, it became the second leading cause of death worldwide [1]. Beyond its health impact, cancer also imposes a growing economic burden with the global cost between 2020 and 2050 projected to reach $25.2 trillion [2]. Despite significant advancements in targeted therapy, chemotherapy, and immunotherapy, major clinical limitations persist, particularly in the form of drug resistance, off-target toxicity, and tumor heterogeneity [3]. These challenges present the need for alternative therapeutic strategies that target cancer’s core vulnerabilities.

One such vulnerability is metabolic reprogramming, now recognized as a hallmark of cancer. Tumor cells undergo profound shifts in energy metabolism to support rapid proliferation and survival under hypoxic or nutrient-deprived conditions. A prime example is the Warburg effect, in which cancer cells preferentially utilize glycolysis even in the presence of oxygen, generating ATP less efficiently but enabling biosynthetic flexibility [4,5]. Furthermore, clinical data suggest strong correlations between metabolic diseases, such as obesity, type 2 diabetes, and dyslipidemia, and increased cancer risk, mediated through chronic inflammation, oxidative stress, and insulin resistance [6]. These findings highlight the therapeutic potential of targeting tumor metabolism as a broadly applicable strategy across diverse cancer types.

At the core of cellular energy regulation lies AMP-activated protein kinase (AMPK), a highly conserved serine/threonine kinase that acts as a master regulator of metabolic balance. AMPK senses energy stress through changes in the AMP: ATP ratio and responds by inhibiting ATP-consuming anabolic processes (e.g., lipid and protein synthesis) while activating catabolic pathways (e.g., fatty acid oxidation and autophagy) [7].

In glucose metabolism, AMPK directly activates phosphofructokinase (PFK)-2, leading to an increased cellular concentration of fructose-2,6-bisphosphate (F-2,6-BP) [8]. F-2,6-BP is a potent allosteric activator of PFK-1, a rate-limiting enzyme and the first committed step of glycolysis [9]. Additionally, AMKP stimulates glycogen breakdown by inducing the fusion of glucose transporter 4 (GLUT4) vesicles with the plasma membrane and activating glycogen phosphorylase, thereby mobilizing stored glucose for immediate energy needs [10]. Furthermore, AMPK inhibits transcription factors, which are required for expression of gluconeogenic enzymes [11].

In fatty acid oxidation, AMPK inactivates acetyl-CoA carboxylase (ACC), the enzyme responsible for the synthesis of malonyl-CoA [12]. Since malonyl-CoA allosterically inhibits carnitine palmitoyltransferase 1 (CPT1)—the transporter of long-chain acyl-CoA into mitochondria—AMPK indirectly facilitates the import of long-chain acyl-CoA into mitochondria [13]. Beyond this role, AMPK regulates triglyceride hydrolysis by phosphorylating hormone-sensitive lipase and suppresses triglyceride esterification by inhibiting mitochondrial glycerol-3-phosphate acyltransferase [14]. This combined action leads to an increased release of free fatty acids into circulation from adipose tissue and enhances fatty acid oxidation in muscle.

In protein metabolism, AMPK inhibits protein biosynthesis by targeting key components of the translation initiation pathway (e.g., tuberous sclerosis complex 2 [TSC2], raptor, transcription initiation factor 1A.66, and eukaryotic elongation factor [eEF] 2 kinase) [15]. It can also induce a shift from cap-dependent translation to cap-independent translation, a more energy-efficient mechanism [16]. Furthermore, AMPK activation strongly suppressed the activity of the master regulator of growth, mammalian target of rapamycin complex 1 (mTORC1). Inhibition of mTORC1 leads to the activation of unc-51-like autophagy-activating kinase 1 (ULK1), a kinase essential for autophagy induction [17].

Overall, AMPK-mediated metabolic shifts not only restore homeostasis but also suppress oncogenic pathways and promote tumor cell death under energetic stress [18,19].

**Figure 1:**
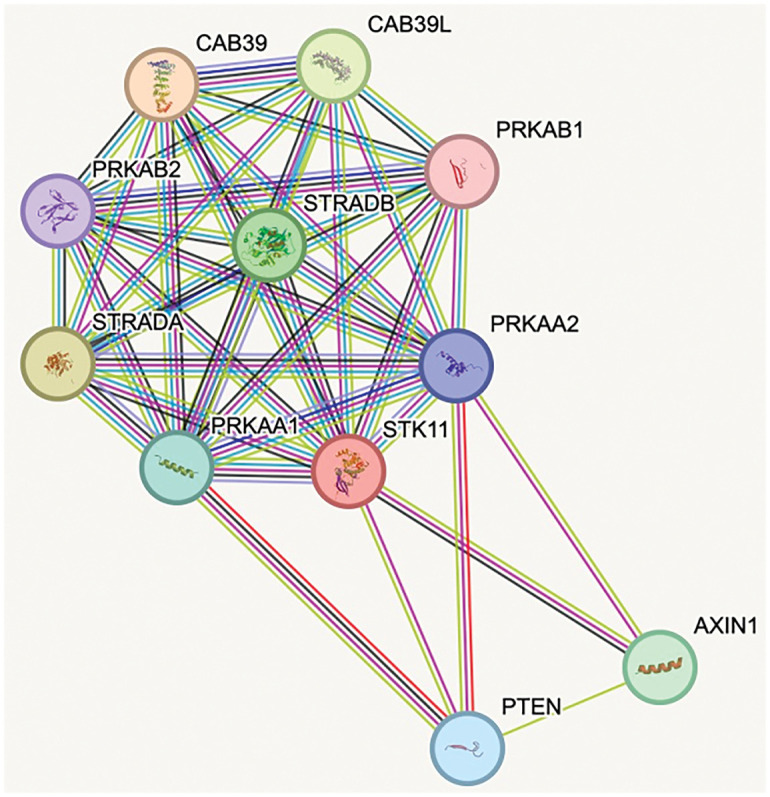
STRING analysis [26]: Protein-protein interaction network highlighting the associations between *STK11* (LKB1) and AMPK subunits. The network illustrates direct interactions between *STK11* and the AMPK α-subunits *PRKAA1* and *PRKAA2*, as well as the β-subunit *PRKAB1*. Each protein is represented as a node, and the connecting lines indicate known or predicted interactions. CAB39, calcium-binding protein 39; CAB39L, calcium-binding protein 39-like; PRKAB1, 5^′^-AMP-activated protein kinase subunit beta-1; PRKAB2, 5^′^-AMP-activated protein kinase subunit beta-2; PRKAA1, 5^′^-AMP-activated protein kinase catalytic subunit alpha-1; PRKAA2, 5^′^-AMP-activated protein kinase catalytic subunit alpha-2; PTEN, phosphatase and tensin homolog; STK11, serine/thereonine kinase 11; STRADA, STE20-related kinase adaptor alpha; STRADB, STE20-related kinase adaptor beta (Source: https://string-db.org)

**Figure 2:**
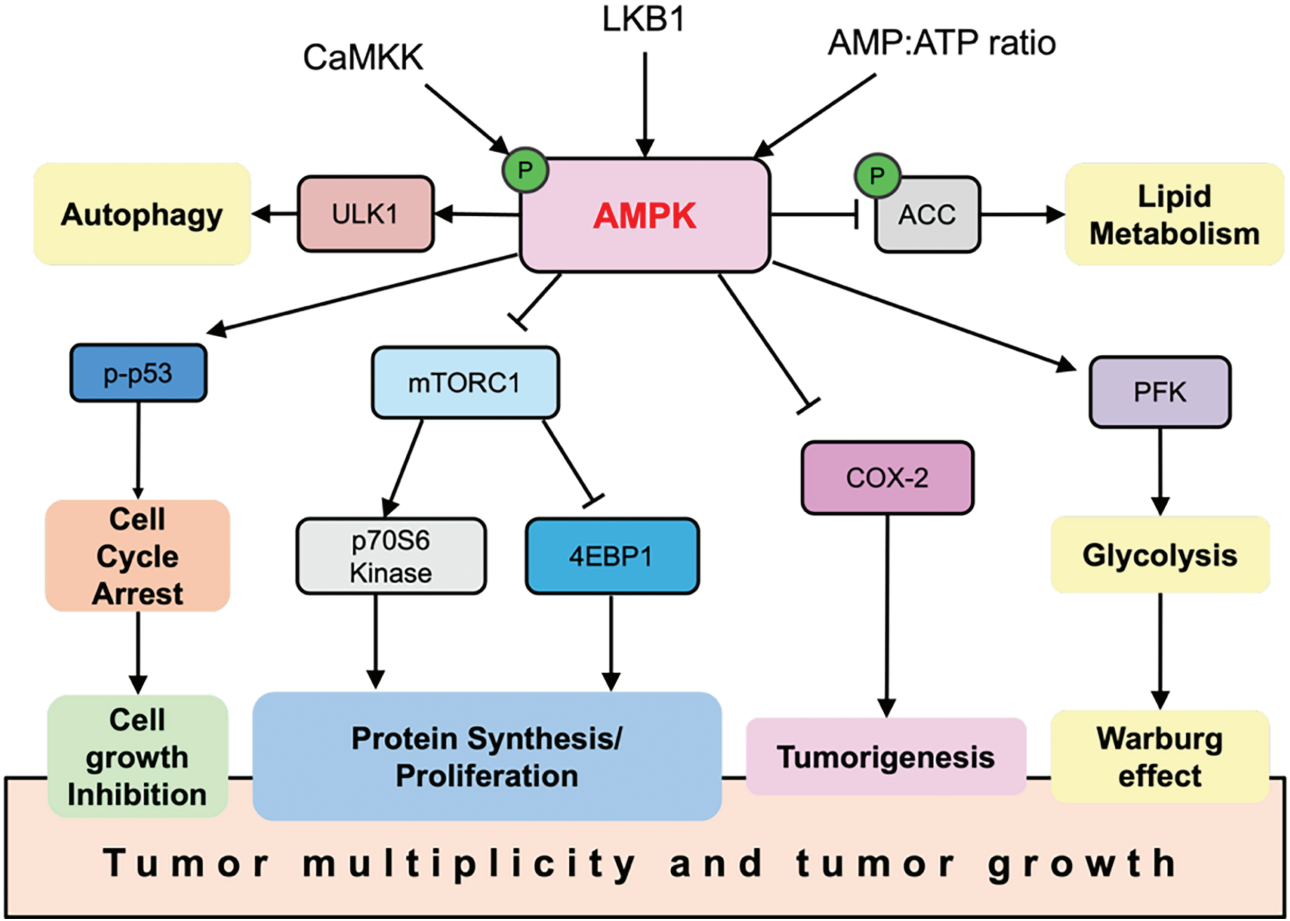
AMPK functions as a tumor suppressor. When activated, AMPK regulates multiple pathways that contribute to its anti-cancer effects. Arrows indicate activation of molecular targets, while bars represent inhibition. 4EBP1, Eukaryotic translation initiation factor 4E-binding protein 1; CaMKK, Calcium/calmodulin-dependent protein kinase kinase 2

Structurally, AMPK is a heterotrimeric complex comprising α (catalytic), β (scaffold and carbohydrate-binding module), and γ (nucleotide-sensing) subunits [20,21]. The liver kinase B1 (LKB1)–AMPK axis is a primary signaling route for AMPK activation, with LKB1 directly phosphorylating AMPK at Thr172, a modification essential for its full activity [22] (Fig. 1). Once activated, AMPK can inhibit tumor-promoting pathways, notably mTORC1, thereby reducing cell proliferation and biosynthetic activity. In addition, AMPK suppresses cyclooxygenase-2 (COX-2), a pro-inflammatory enzyme associated with tumorigenesis, and promotes the phosphorylation of the tumor suppressor p53, leading to cell cycle arrest (Fig. 2). Loss of LKB1 is frequently observed in several cancer types and is associated with AMPK inactivation, metabolic dysregulation, and enhanced tumor progression [23]. Notably, tumors with LKB1 loss are more vulnerable to AMPK-inducing agents, while LKB1-wildtype tumors may resist such stress, suggesting a context-dependent therapeutic window [24,25]. Thus, restoring or enhancing AMPK activity via the LKB1 pathway represents a rational therapeutic goal in metabolically dysregulated tumors.

Various pharmacological AMPK activators—such as metformin [27,28], phenformin [29], and canagliflozin [30,31]—have been studied in both preclinical and clinical settings. These agents induce AMPK activation indirectly through mitochondrial inhibition or glucose modulation and have shown potential in select cancer subtypes. However, their clinical efficacy is limited by factors such as low tumor-specific bioavailability, systemic side effects, and variability in AMPK-dependence among tumor types [3,32]. These challenges highlight the need for more precise and sustained activation methods that selectively target cancer metabolism without affecting normal tissues. Enhancing AMPK signaling within tumor cells will help reprogram aberrant metabolism, inhibit tumor growth, and augment existing therapeutic regimens.

This review will examine the central role of AMPK in tumor metabolism, critically assess the clinical landscape of AMPK-targeted therapies.

## 2 AMPK Activation as a Therapeutic Strategy in Cancer

### 2.1 Molecular Mechanisms Underlying the Antitumor Effects of AMPK

AMPK is a master regulator of cellular energy homeostasis, and its activation in tumor cells acts as a countermeasure against the metabolic reprogramming characteristic of cancer, commonly referred to as the Warburg effect [33]. Upon activation, AMPK conserves energy by shutting down energy-consuming anabolic processes. Specifically, it phosphorylates and inactivates ACC1 and ACC2, thereby halting fatty acid synthesis, and also targets 3-hydroxy-3-methylglutaryl coenzyme A (HMG-CoA) reductase to suppress the cholesterol/mevalonate biosynthetic pathway [34]. These changes deprive tumor cells of critical lipids and building blocks required for rapid proliferation. Additionally, AMPK inhibits the growth-promoting kinase mTORC1 through phosphorylation of TSC2 or raptor, leading to reduced protein synthesis and suppressed glycolytic flux. Collectively, these actions induce a metabolic shift away from the high-glucose, high-biosynthesis state of tumor cells toward a more quiescent phenotype effectively reversing the Warburg effect [35]. This energy stress, combined with mTORC1 inhibition, ultimately slows tumor cell growth and may even trigger an energy crisis that compromises cancer cell viability.

In addition to its role in tumor metabolism, AMPK also exerts antitumor effects by modulating immune cell function, particularly in T cells [36]. T cell-mediated responses are central to effective tumor immunosurveillance [37,38]; however, in the nutrient-depleted tumor microenvironment (TME), T cells face intense metabolic competition with tumor cells, especially for glucose. Glucose limitation impairs T cell activation and induces expression of inhibitory receptors such as programmed cell death protein 1 (PD-1), driving metabolic exhaustion and a shift from glycolysis to fatty acid oxidation [36]. Furthermore, immunosuppressive cytokines like interleukin (IL)-10 can suppress glycolytic metabolism in T cells through AMPK activation. While such activation may, in some cases, attenuate effector T cell responses, accumulating evidence suggests a supportive role for AMPK in sustaining T cell function under metabolic stress. For example, mice lacking AMPKα1 in T cells exhibit accelerated tumor growth [39], whereas treatment with metformin [31,40]; an indirect activator of AMPK has been shown to enhance CD8^+^ T cell infiltration and promote tumor rejection, particularly when used in combination with PD-1 checkpoint blockade. These findings highlight AMPK’s potential in maintaining T cell metabolic fitness and bolstering antitumor immunity within the hostile conditions of the TME [36].

AMPK also exerts its regulatory effects on ion channels and transporters in cancer cells [41]. It can directly phosphorylate various transport proteins, thereby altering their function, activity and cellular localization. For instance, AMPK-mediated direct phosphorylation inhibits the activity of cystic fibrosis transmembrane conductance regulators (CFTR), a chloride channel; reduces the activity of the epithelial sodium channel (ENaC); and slows the inactivation of the voltage-gated sodium channel Nav1.5 [41]. In addition, AMPK downregulates several potassium channels through direct phosphorylation, including Ca2^+^-activated potassium channels (e.g., KCa3.1), inwardly rectifying potassium channels (e.g., Kir1.1, Kir2.1), and voltage-gated potassium channels (e.g., Kv1.5, Kv2.1, Kv7.1, Kv11) [42]. Beyond direct phosphorylation, AMPK also modulates transport protein indirectly through several mechanisms: stimulation of ubiquitin ligase, inhibition of Rab GTPase activating protein (GAP) tre-2/BUB2/cdc 1 domain family (TBC1D1), stimulation of phosphatidylinositol 3-phosphate 5-kinase (PIKfyve), inhibition of Phosphatase and tensin homolog (PTEN) via glycogen synthase kinase 3β (GSK3β), F-actin modulation, and downregulation of transcription factor NF-κB [42]. Through these pathways, AMPK’s regulation of ion channels and transporters has complex, context-dependent effects on cancer cell behaviors—including proliferation, apoptosis, migration, and multidrug resistance—by modulating metabolic pathways, autophagy, and ABC transporter expression.

One of the most clinically significant aspects of AMPK activation is its ability to enhance tumor sensitivity to conventional therapies such as chemotherapy and radiation. By suppressing DNA repair mechanisms and pro-survival signaling, AMPK activation lowers the threshold for therapy-induced cancer cell death. As a result, co-treatment with AMPK activators and traditional anti-cancer agents has demonstrated synergistic effects in various preclinical models [34]. For instance, 5-Aminoimidazole-4-carboxamide ribonucleoside (AICAR)-induced AMPK activation sensitizes colorectal cancer cells to 5-fluorouracil (5-FU), leading to greater apoptotic responses than 5-FU alone [43]. In pancreatic cancer, metformin enhances the efficacy of gemcitabine, likely by altering tumor metabolism and affecting the tumor stroma [44]. Similarly, AMPK activators such as phenformin and AICAR have been reported to increase tumor radiosensitivity by disrupting cellular energy supply and impairing DNA repair capacity [34]. In essence, AMPK activation generates a metabolically unfavorable environment for tumor survival under therapeutic stress: it suppresses growth, promotes apoptosis, and diminishes the tumor’s ability to recover post-treatment. These insights have led to growing interest in repurposing AMPK activators like metformin as adjuvant therapies to improve treatment outcomes in cancers such as colorectal, breast, and lung cancer.

In cancer therapy, the activation of AMPK plays a crucial role in disrupting tumor proliferation and metabolic plasticity [45]. Various agents, including metformin, phenformin, salicylate, berberine, resveratrol, and quercetin have been explored for their ability to activate AMPK and exert anti-cancer effects (Table 1).

**Table 1:** Notable Clinical Investigations of AMPK-Targeted Cancer Therapies

Cancer	Method	Phase	Trial identifier	Ref.
ALL	Metformin	NA	NCT03118128, NCT05326984	[81,82]
Bladder	Metformin, simvastatin	II	NCT02360618	[83]
Breast	Metformin	II	NCT01266486	[84]
Breast (metastatic)	Lapatinib, metformin	II	NCT01477060	[85]
Breast neoplasms, lung neoplasms, liver, lymphoma, kidney	Metformin, delayed metformin, sirolimus	I	NCT02145559	[86]
CLL (relapsed)	Metformin	II	NCT01750567	[87]
Colon, pancreas, lung	Vitamin C	II	NCT03146962	[88]
DLBCL	Metformin, rituximab, cyclophosphamide, doxorubicin, vincristine, prednisone, pegfilgrastim	II	NCT02531308	[89]
Endometrium	Metformin	Early I	NCT01911247	[90]
	Metformin	II	NCT02042495	[91]
Endometrium, Ovary	Letrozole, abemaciclib, LY3023414, metformin, zotatifin	II	NCT03675893	[92]
Kidney	Sunitinib + isoquercetin,	I, II	NCT02446795	[93]
Leukemia	Midostaurin	II	NCT00233454	[48,49, 94]
Liver	Resveratrol	I, II	NCT02261844	[95]
Lung	Metformin, carbohydrate-restricted diet	II	NCT02019979	[96]
Melanoma (Skin)	Aspirin	II	NCT04062032	[97]
Melanoma (metastatic)	Metformin	II	NCT01840007	[98]
NSCLC	Metformin hydrochloride	III	NCT05445791	[99]
Oral cavity neoplasm	Metformin hydrochloride/pioglitazone hydrochloride extended-release tablet	II	NCT02917629	[100]
Pancreas	Everolimus + octreotide LAR + metformin	II	NCT02294006	[101]
	Gemcitabine, erlotinib, metformin,	II	NCT01210911	[102]
Prostate	Abiraterone acetate, abiraterone acetate + degarelix, degarelix	II	NCT01751451	[103]
	Metformin	II	NCT00881725, NCT01433913, NCT01620593	[104–106]
	Metformin, radiation	IV	NCT02511665	[107]
Thoracic neoplasm	Metformin	Early I	NCT03477162	[108]

Note: ALL, acute lymphoblastic leukemia; DLBCL, diffuse large B cell lymphoma; NSCLC, non-small cell lung cancer; NA, Not Available.

### 2.2 AMPK Activators Proposed in Cancer Therapy

#### 2.2.1 Biguanides

Interestingly, a variety of anti-diabetic drugs are being tested for their potential use in cancer therapy beyond their primary use. Biguanides, particularly metformin and phenformin, are being explored in cancer therapy for their potential to inhibit tumor growth by altering cellular metabolism and activating AMPK. Metformin indirectly activates AMPKα1/α2 by inhibiting mitochondrial Complex I [27]. This activation leads to the suppression of protein prenylation and an increase in reactive oxygen species (ROS), culminating in the downregulation of p70 S6 kinase (p70S6K) and eIF4E-binding protein 1 (4EBP1), critical downstream effectors of the mTOR pathway [46]. In acute myeloid leukemia (AML), metformin induces G0/G1 arrest mediated by p21/p27 and reduces levels of cyclin-dependent kinase (CDK) 4 and cyclin D1 [47–49]. In breast cancer, particularly human epidermal growth factor receptor (HER2)-positive and triple-negative breast cancer (TNBC) subtypes, both metformin and phenformin are found to suppress tumor growth and metastasis by downregulating c-MYC and reducing invasiveness through the suppression of hyaluronan synthase 2 (HAS2) [50–52]. Furthermore, phenformin enhances apoptosis in bladder cancer [53,54] and increases vulnerability in glioblastoma [55] and hepatocellular carcinoma [56,57] via AMPK-mediated nutrient deprivation. Phenformin also enhances non-small cell lung cancer (NSCLC) radiosensitivity by impairing DNA repair mechanisms [58]. However, phenformin was removed from most markets in the 1970s due to a high risk of fatal lactic acidosis [59].

#### 2.2.2 Non-Biguanide Anti-Diabetic Drugs

Non-biguande anti-diabetic drugs, such as canagliflozin and pioglitazone, have also been tested in cancer therapy. Canagliflozin, an oral antidiabetic drug that inhibits sodium-glucose co-transporter 2 (SGLT2), inhibits mitochondrial Complex I, thereby inducing oxidative stress and promoting cancer cell death. This mechanism enhances the efficacy of radiotherapy, positioning canagliflozin as a potential adjunct in cancer treatment strategies [30]. Pioglitazone, a peroxisome proliferator-activated receptor gamma (PPARγ) agonist, exhibits anti-proliferative and pro-differentiation effects in several cancer models. It is also currently under investigation for its interaction with AMPK and therapeutic synergy with metformin. This combination has been shown to suppress desmoplasia and stromal support by inhibiting pancreatic stellate cell activation and downregulating fibrogenic cytokines [27,60].

#### 2.2.3 Synthetic mTORC1 Inhibitors

Several anti-cancer and immunomodulatory agents were developed targeting mTORC1. The combination of everolimus and octreotide long-acting release (LAR) has demonstrated clinical benefits in neuroendocrine tumors (NETs) through effective inhibition of mTORC1 signaling [61]. Everolimus directly targets the phosphoinositide 3-kinase (PI3K)/AKT/mTOR pathway to suppress tumor growth and proliferation. Octreotide is a synthetic analog of somatostatin, a hormone that inhibits the release of several other hormones and is used primarily for its ability to reduce the symptoms of hormone-secreting tumors [62]. Additional multi-cancer agents such as LY3023414, a PI3K/mTOR inhibitor; zotatifin, an eIF4A inhibitor affecting translation; and sirolimus, which inhibits mTORC1 downstream of AMPK activation, are being explored [63–65].

#### 2.2.4 Natural Compounds

Natural compounds, artificially synthesized but originally found in nature, are widely considered for their potential roles in cancer prevention and treatment due to their diverse biological activities and relatively low toxicity [66]. Berberine, another plant-derived compound, reduces the viability of colon and lung cancer cells by inducing apoptosis and activating caspase-3 and the miR-21/programmed cell death protein 4 (PDCD4) axis associated with tumor progression [67–69]. Resveratrol, a natural compound found in the skin of grapes and associated with red wine, is an AMPK activator extensively studied for its cancer-preventive properties in clinical trials and preclinical models [70]. Quercetin, a flavonoid found in many fruits and vegetables, inhibits mTOR and reduces glycolysis through AMPK, inhibiting tumor proliferation and inducing apoptosis [71–73]. In colorectal cancer cells, quercetin increases p-AMPK levels and downregulates cyclin D1 and the anti-apoptotic Bcl-2 [74,75]. Isoquercetin, studied for melanoma, indirectly activates AMPKα, contributing to angiogenesis inhibition and apoptosis [76]. Furthermore, vitamin C has been shown to activate AMPK by disrupting glyceraldehyde 3-phosphate dehydrogenase (GAPDH) and inducing oxidative stress, particularly in KRAS/BRAF-mutant tumors [77].

#### 2.2.5 Non-Canonical Inhibitors of AMPK

Antifungal medication itraconazole has been found to indirectly activate AMPKα1 by inhibiting mTOR, beyond its antifungal properties, and is in Phase II trials for NSCLC [78]. In liver cancer, simvastatin, a cholesterol-lowering agent, contributes to its role in lipid regulation by modulating the AMPK-mTOR axis [61]. Additionally, NSCLC treatments involve erlotinib, which targets the epidermal growth factor receptor (EGFR) pathway and intersects with AMPK-regulated metabolism [79]. Together, these agents highlight the diversity of strategies leveraging AMPK pathways in cancer therapy, from metabolic reprogramming to immunomodulation and cytotoxic synergy.

## 3 Current Clinical Development of AMPK Activation in Anti-Cancer Therapy

AMPK activators have shown promise in preventing tumor growth and migration. Many of these activators have been approved to treat cancers [80]. Moreover, several AMPK activators are currently undergoing preclinical or clinical trials as potential anti-cancer drugs. Existing drugs that activate AMPK are also being repurposed for cancer treatment, highlighting ongoing research and development in clinical trials (Table 1).

### 3.1 Hepatobiliary and Digestive System

#### 3.1.1 Colorectal Cancer (CRC)

Recent trials in CRC have explored whether targeting metabolism can improve patient outcomes [109]. One notable approach involves exercise: the Phase 2 EDICT trial investigated whether an exercise program in CRC patients could trigger AMPK-related metabolic changes that might slow tumor growth [110]. While results are still pending, this reflects growing interest in non-pharmacologic strategies to activate AMPK [110].

Another promising avenue is the use of aspirin, which indirectly activates AMPK by inhibiting prostaglandin synthesis. Long-term follow-ups of the CAPP trials and findings from the recent Phase 3 ALASCCA trial provide evidence that aspirin can reduce CRC recurrence in certain molecularly defined patient groups. For instance, in the ALASCCA trial, adjuvant aspirin therapy in patients with stage III CRC and PIK3CA-mutated tumors halved the 3-year recurrence risk compared to placebo [111]. This suggests that targeting the PI3K–AMPK–mTOR axis with a repurposed drug like aspirin offers a meaningful benefit in a subset of CRC patients.

Metformin has mostly been evaluated in observational settings, with some reports indicating that CRC patients with diabetes on metformin have better outcomes. However, dedicated trials are limited. One small Phase 1 study combined metformin with high-dose vitamin C, which induces ROS, in KRAS-mutant colorectal and pancreatic cancers. This was based on preclinical evidence that vitamin C can selectively kill KRAS-mutant cells by inducing metabolic stress [88]. Early results showed some tumor metabolic responses, but no definitive efficacy yet.

Overall, in CRC, the most impactful metabolic intervention to date is aspirin in the adjuvant setting for genetically selected patients, while trials of metformin remain exploratory. Current research is increasingly focused on integrating metabolic therapies with molecular profiling, such as applying AMPK activators or metabolic diets in patients with obesity, insulin resistance, or specific mutations that confer metabolic vulnerabilities.

#### 3.1.2 Liver Cancer

Hepatocellular carcinoma (HCC) often develops in the context of diabetes and nonalcoholic fatty liver disease (NAFLD), positioning metformin as an attractive candidate for both chemoprevention and therapy. Epidemiological studies have indicated that metformin use is associated with a significantly lower risk of developing HCC in diabetic patients [112]. However, clinical trials directly targeting AMPK in HCC have been limited.

A planned Phase 1/2 trial of resveratrol—a nutraceutical that activates AMPK and sirtuins—aimed to assess its effects on liver tumor cell growth and metabolism [86,95] but was withdrawn before enrollment, likely due to feasibility challenges [95]. Consequently, no clinical data on resveratrol in liver cancer were collected [95]. Another small pilot study combined metformin with transarterial chemoembolization (TACE) in HCC, but the results were inconclusive regarding tumor response, and concerns about lactic acidosis limited metformin use in cirrhotic patients [86].

Due to a lack of interventional trial data in liver cancer, current insights come mostly from retrospective analyses. These suggest metformin might improve survival in HCC patients, particularly those receiving immune checkpoint inhibitors, by modifying TME [113]. There is also evidence that metformin, when used after curative HCC treatment like surgery or ablation, may reduce recurrence rates. Nevertheless, HCC is a complex disease where liver function and hepatitis status can confound outcomes, making it challenging to isolate the effect of AMPK activation [114–116].

In summary, while liver cancer is biologically amenable to AMPK-targeting strategies, their clinical application remains largely theoretical. Ongoing preventative trials in high-risk patients, such as those with nonalcoholic steatohepatitis (NASH) with fibrosis, will determine if metformin can lower HCC incidence. For active HCC therapy, future studies might explore combining metformin or aspirin with newer systemic treatments like anti-PD-1 immunotherapy or tyrosine kinase inhibitors to assess potential additive benefits.

#### 3.1.3 Pancreatic Cancer

Despite strong epidemiological links, prospective trials of AMPK activators in pancreatic ductal adenocarcinoma (PDAC) have largely yielded disappointing. A notable Phase 2 trial in advanced pancreatic cancer tested the addition of metformin to standard gemcitabine plus erlotinib therapy [102]. Unfortunately, metformin did not improve six-month survival or median overall survival (OS) [102]. Similarly, other trials in metastatic disease—including combinations of metformin with FOLFIRINOX chemotherapy—were negative, leading investigators to conclude that conventional-dose metformin offers no survival advantage in unselected pancreatic cancer patients [102].

A Phase 2 randomized clinical trial in the Netherlands also assessed the addition of metformin to standard chemoradiation in locally advanced pancreatic cancer. The study found no improvement in OS, resulting in early termination of the trial due to futility [117]. These results suggest that pancreatic tumors—often characterized by hypoxia and dense desmoplasia—may not respond to indirect metabolic interventions like metformin unless patients have metabolic profiles, such as hyperinsulinemia.

There is, however, ongoing interest in combining AMPK activators with targeted therapies for well-differentiated NETs. The Phase 2 MetNET-1 trial is currently evaluating the combination of everolimus, octreotide, and metformin in advanced pancreatic NETs [101], aiming to enhance mTOR pathway blockade via AMPK activation. While final results are pending, a prior single-arm study of metformin monotherapy in 28 patients with metastatic NET reported a six-month disease control rate of 46%, but no objective tumor responses and a median progression-free survival (PFS) of approximately 6.2 months, essentially equivalent to historical placebo rates [101]. This suggests metformin alone has minimal activity in NETs. Combination strategies may offer better tumor suppression, as hinted by retrospective data showing that diabetic NET patients receiving metformin and everolimus experienced longer PFS [101].

In summary, the consensus for pancreatic malignancies is that metformin alone is ineffective for the general patient population. Future trials are focusing on specific niches—pairing phenformin or metformin with immunotherapy or targeting patients with metabolic syndrome—in hopes of identifying subgroups that might derive meaningful clinical benefit.

### 3.2 Reproductive System

#### 3.2.1 Breast Cancer

Large later-phase trials have investigated the use of metformin in the adjuvant setting of breast cancer. The Phase 3 MA.32 trial, which included over 3600 patients, found that adding metformin to standard therapy did not improve invasive disease-free survival or OS in early breast cancer. This result was consistent for both hormone receptor–positive and TNBC subsets, leading researchers to conclude that metformin should not be used as adjuvant therapy in unselected breast cancer patients [118].

Short-term preoperative “window” studies have demonstrated some biological effects. For example, a UK Phase 2 trial of 2–3 weeks of neoadjuvant metformin observed changes in tumor metabolism and increased tumor fluorodeoxyglucose (FDG) uptake, though there was variability in metabolic responses between tumors [119]. Some neoadjuvant studies also reported modest reductions in the proliferation marker Ki-67 with metformin treatment, although these findings were not always consistent [120].

In HER2-positive metastatic breast cancer, metformin has been combined with therapies like lapatinib and endocrine treatment in Phase 2 trials, but without definitive efficacy signals; the results have been mixed or not significant [84]. In the case of TNBC, there is considerable interest in targeting metabolic vulnerabilities, although few trials of AMPK activators have been completed [84,85]. Other metabolic interventions have shown promise: notably, the addition of carboplatin to standard neoadjuvant chemotherapy significantly improved event-free and overall survival in TNBC. A Phase 3 trial, involving 720 patients, achieved higher pathologic response rates and established platinum-based chemotherapy as a standard treatment in TNBC [121]. While not directly targeting AMPK, this highlights the importance of targeting cancer metabolism/stress in TNBC [121].

Ongoing studies are exploring metformin in combination with standard neoadjuvant chemotherapy or immunotherapy in TNBC. However, as of now, no Phase 3 data have shown a clear benefit of metformin in this subtype [121]. In summary, despite a strong preclinical rationale, metformin’s clinical impact in breast cancer has been limited. Future approaches may require better biomarkers, such as tumor metabolic profiles or insulin levels, to identify patient subsets that are more likely to respond.

#### 3.2.2 Endometrial and Ovarian Cancers

Endometrial carcinoma is closely associated with obesity and hyperinsulinemia, making AMPK activation an appealing therapeutic strategy. Small preoperative trials have demonstrated that metformin can modulate tumor biology in endometrial cancer [122]. In an early-phase window study, obese endometrial cancer patients took metformin for a short period before surgery. The tumors exhibited an average decrease of 11%–15% in the Ki-67 proliferation index and significant inhibition of the mTOR pathway signaling, indicated by reduced levels of p-AKT, p-S6, and p-4EBP1 [91]. Patients who responded metabolically to metformin showed serum changes, such as increased lipolysis, fatty acid oxidation, suggesting that metformin’s antitumor effect was linked to systemic metabolic improvements [91]. These findings support further investigation of metformin in endometrial cancer, particularly in metabolically abnormal patients. Although a Phase 2 trial was planned to administer metformin pre-surgery and measure changes in tumor pS6 and other AMPK/mTOR biomarkers, it was withdrawn before completion possibly due to slow accrual [91].

Ongoing Phase 2 studies are exploring metformin in combination with other treatments. The RESOLVE trial is testing a three-drug combination in recurrent endometrial and ovarian cancers: letrozole, abemaciclib (a CDK4/6 inhibitor), with or without the addition of metformin [92]. The rationale is that metformin’s insulin-lowering and AMPK-activating effects might enhance hormone therapy and CDK4/6 inhibition, especially in obese or insulin-resistant patients with endometrial cancer [92]. Another approach in ovarian cancer was evaluated in a Phase 2 study in advanced-stage disease, where neoadjuvant chemotherapy was combined with metformin [92]. The metformin-treated cohort showed promising results, indicating a higher than expected 18-month relapse-free survival (~59%) and a significant reduction in ALDH^+^CD133^+^ cancer stem cells in tumors. Although that was a single-arm translational study, it suggested that metformin may improve chemotherapy responses and alter TME [92]. Retrospective data for ovarian cancer have also hinted at improved survival in diabetic patients on metformin during chemotherapy.

Currently, no Phase 3 trials have definitively proven metformin’s benefit in gynecologic cancers, but the wealth of Phase 2 data suggests it is well-tolerated and potentially beneficial when combined with other therapies. Recent preclinical studies have highlighted the potential of metformin to enhance antitumor immunity and overcome immune evasion in ovarian cancer by modulating the tumor microenvironment and augmenting the efficacy of programmed death-ligand 1 (PD-L1) blockade [123]. Future larger trials may focus on obese diabetic patients or use metformin as a maintenance therapy to delay recurrence.

#### 3.2.3 Prostate Cancer

Prostate cancer has been the focus of several Phase 2 trials investigating metformin, but the results have been largely negative. In a randomized pre-prostatectomy trial, patients received metformin for approximately one month before surgery to assess its effects on prostate tissue. While metformin was detectable at measurable levels within the prostate, it did not significantly change tumor cell proliferation, apoptosis, or mTOR pathway markers compared to placebo [106]. Similarly, serum insulin, insulin-like growth factor 1 (IGF-1), and other systemic biomarkers remained largely unchanged over this short treatment period, suggesting no immediate antitumor effects.

A larger Phase 3 trial evaluated metformin in patients on active surveillance for low-risk prostate cancer. The findings showed that metformin failed to slow cancer progression, with similar rates of biopsy progression to higher-grade disease or need for treatment in both the metformin vs. placebo groups. Notably, an exploratory subgroup analysis suggested that men with higher body mass index (BMI) on metformin had slightly worse progression outcomes than those on placebo, although the sample size was small. Additionally, metformin’s known side effects—mainly gastrointestinal upset—were common, with about 30% of men experiencing gastrointestinal discomfort or fatigue [106]. This trial, presented in 2024, provided strong (level 1) evidence that metformin does not benefit early prostate cancer, dampening enthusiasm for its off-label use in this setting.

Metformin has also been studied in advanced prostate cancer, particularly in combination with hormonal therapies. One randomized Phase 2 trial examined adding metformin to androgen deprivation therapy in hormone-sensitive prostate cancer, but results were inconclusive. In castration-resistant prostate cancer, a Swiss Phase 2 pilot trial (MetAb-Pro) tested metformin in combination with ongoing abiraterone therapy for men who had prostate-specific antigen (PSA) progression on abiraterone [104]. The primary endpoint—disease control at 12 weeks—was achieved in only 12% of patients, falling below the predefined threshold for efficacy [104]. Nearly all patients continued to experience PSA progression despite metformin, indicating no meaningful clinical benefit. Although toxicity was generally mild, one patient had to stop metformin due to diarrhea.

These findings align with larger observational analyses suggesting that while metformin (and sometimes concurrent statins) may appear to improve outcomes in men on abiraterone or enzalutamide, prospective confirmation is lacking [103]. Overall, the available evidence indicates that although metformin is safe to combine with prostate cancer treatments, it does not meaningfully enhance their efficacy in most patients. As a result, ongoing trials have become scarce, and research attention has shifted to other metabolic targets, such as targeting obesity-related inflammation, and incorporating exercise and diet interventions for prostate cancer management.

### 3.3 Urinary System

#### 3.3.1 Bladder Cancer

Bladder cancer is another malignancy associated with metabolic syndrome, attracting interest in repurposing metabolic drugs for its treatment. A Phase 2 trial in non-muscle-invasive bladder cancer (NMIBC) is currently evaluating the combination of metformin and simvastatin during intravesical therapy [83]. The aim is to determine whether dual AMPK activation by metformin and inhibition of cholesterol pathway by statins can reduce tumor cell proliferation and recurrence. The primary outcome being measured is the tumor proliferation rate during therapy [83]. As of now, no efficacy results have been reported.

There is some indirect clinical evidence supporting this approach: retrospective studies have shown that diabetic bladder cancer patients on metformin have lower recurrence rates, and statin use has been associated with improved outcomes in bladder cancer. This makes the combination of metformin and simvastatin particularly intriguing [83]. However, it is worth noting that a previous Phase 2 trial involving metformin for muscle-invasive bladder cancer was closed early due to a lack of benefit [83]. Therefore, while AMPK activators are being tested in bladder cancer, their role remains unproven. The ongoing trial will provide insights into whether metabolic manipulation can enhance standard bladder cancer treatments or prevent disease progression.

#### 3.3.2 Renal Cell Carcinoma

In renal cell carcinoma (RCC), a Phase 1/2 trial has evaluated the addition of isoquercetin to first-line sunitinib for metastatic RCC [93]. Sunitinib, a vascular endothelial growth factor (VEGF)-targeted therapy, is the standard of care, and the hypothesis is that isoquercetin’s antioxidant and AMPK-activating properties could enhance antitumor activity using positron emission tomography (PET)-based metabolic response, response evaluation criteria in solid tumors (RECIST), and patient-reported quality of life outcomes [93]. Although results are not yet available, this study reflects a broader strategy of combining metabolic therapies with targeted agents in RCC.

While there have been no large prospective trials of metformin specifically in RCC, observational studies suggest that diabetic patients on metformin may experience prolonged survival when receiving immunotherapy or targeted therapies for kidney cancer [114]. Preclinical studies further support the rationale, showing that AMPK activation can inhibit renal carcinoma cell growth and mTOR signaling, particularly in tumors with metabolic dysregulation [124]. In clinical practice, some physicians have occasionally used metformin off-label for insulin-resistant RCC patients, though without strong supporting evidence [125,126]. The ongoing isoquercetin study and upcoming trials investigating other AMPK activators, such as phenformin, are expected to clarify whether metabolic modulation offers a meaningful benefit in RCC [93]. For now, standard treatments—including tyrosine kinase inhibitors (TKIs) and immunotherapies—remain the mainstay, with metabolic drugs considered experimental adjuncts.

### 3.4 Respiratory and Upper Aerodigestive System

#### 3.4.1 Lung Cancer

Several Phase 2 trials have tested adding metformin to standard therapy. In one study of metastatic NSCLC, metformin combined with carboplatin/paclitaxel/bevacizumab showed a higher 1-year PFS (47% *vs*. 15% historical control) and a slight OS improvement, suggesting a potential benefit. However, a larger randomized trial in stage III NSCLC found no improvement in 1- or 2-year PFS or OS from adding metformin to chemoradiation. Metformin was well-tolerated in that study but did not enhance outcomes [96,108].

Ongoing Phase 3 research is focusing on specific subsets. For example, the METLUNG trial is evaluating EGFR-mutant NSCLC patients on EGFR tyrosine kinase inhibitors ± metformin (312 patients, Stage IIIB-IV) to see if metformin prolongs PFS [96,99]. Another approach combined metformin with a carbohydrate-restricted diet alongside platinum chemotherapy in advanced NSCLC; this Phase 2 trial was terminated early, underscoring the challenges of dietary interventions [96,127]. Overall, in lung cancer, the clinical efficacy of AMPK activation via metformin remains uncertain; promising signals in small studies have been tempered by larger trials showing no clear benefit, indicating a need for better patient selection or combination strategies.

#### 3.4.2 Head and Neck Squamous Cell Carcinoma (HNSCC)

In head and neck squamous cell carcinoma (HNSCC), metformin has been tested in the preoperative setting. A Phase 2 “window of opportunity” trial tested a combination of metformin and pioglitazone (ACTO plus Met XR) in patients with oral or oropharyngeal squamous cell carcinoma [100]. Patients took the drug combination before surgery to assess biologic effects on the tumor, with the primary endpoint being changes in the tumor Ki-67 proliferation index [100]. Although the trial was terminated early, likely due to poor accrual, it demonstrated the feasibility of short-term metabolic therapy in HNSCC. Additionally, retrospective studies have reported that diabetic head and neck cancers who were metformin during chemoradiation experienced improved locoregional control and survival, suggesting a potential radio-sensitization effect worth further investigating [100]. There is also growing interest in using metformin for oral premalignant lesions, such as dysplasia to prevent progression—based on its capacity to activate AMPK and potentially trigger autophagy in damaged mucosal cells [128]. While still experimental, this represents a promising direction for future cancer prevention strategies.

### 3.5 Integumentary System (Skin)

Metabolic therapy in melanoma has focused more on aspirin rather than metformin [97]. A Phase 2 trial conducted in Utah enrolled individuals at elevated risk for melanoma to receive either low-dose or high-dose aspirin [97]. This completed study primarily assessed aspirin’s effects on systemic metabolism and tissue biomarkers, including AMPK activation in nevi and immune cells [97].

While there have been some case reports and small studies examining metformin use in melanoma, particularly among diabetic patients, no major clinical trials have been conducted [98]. Interestingly, melanoma cells often exhibit high basal AMPK activity, raising questions about whether further AMPK activation would provide therapeutic benefits. Instead, current research interest has shifted toward dietary interventions, such as ketogenic diets or calorie restriction, which may modulate melanoma metabolism and potentially enhance the effects of checkpoint blockade therapies. These strategies remain experimental [129,130].

The key clinical takeaway is that commonly used drugs like aspirin may offer chemo-preventive benefits for high-risk melanoma patients, with a favorable safety profile [98]. If the ongoing trial results demonstrate that aspirin beneficially alters skin or blood biomarkers—for example, by reducing inflammatory mediators or increasing tissue AMPK activation—this could pave the way for larger studies investigating whether aspirin can reduce melanoma incidence or improve patient outcomes.

### 3.6 Immune System

#### 3.6.1 ALL

Early clinical evidence suggests that metformin can improve outcomes in high-risk groups of ALL. A randomized trial in pediatric ALL reported that adding metformin to chemotherapy reduced the relapse risk by approximately 56%. Another Phase 2 study focused on ALL patients with high ATP-binding cassette subfamily B member 1 (ABCB1) expression [81,82]. In this subgroup, the combination of metformin and standard chemotherapy improved survival rates and lowered the incidence of treatment failure and early recurrence, with odds ratios between 0.05 and 0.07 favoring metformin [89]. These findings suggest that metformin may enhance the sensitivity of leukemia cells to chemotherapy, possibly by affecting AMPK and drug transporter pathways.

A pilot Phase 2 trial is currently underway, examining metformin as a therapy to delay disease progression in patients with relapsed or untreated chronic lymphocytic leukemia (CLL), a type of indolent adult leukemia. While results are not yet published, preclinical data support the rationale that metformin targets leukemia cell metabolism and self-renewal [87]. In the context of ALL, the clinical implication is that metformin could serve as a low-toxicity adjunct to enhance chemotherapy efficacy, particularly in patients with chemo-resistant profiles [87]. Future larger trials will be needed to confirm the survival benefits and identify which ALL patients benefit most from AMPK activation therapy.

#### 3.6.2 Lymphomas

Among all cancer types, diffuse large B-cell lymphoma (DLBCL) has provided one of the strongest indications for metformin’s efficacy. In a randomized Phase 2 trial, metformin was added to standard rituximab-cyclophosphamide/hydroxydaunorubicin/oncovin/prednisone (R-CHOP) immunochemotherapy in previously untreated DLBCL patients. The metformin group achieved a remission rate of 92% compared to 74% in the control group receiving R-CHOP alone. Two-year relapse rates were significantly lower with metformin and the 2-year OS was higher [89]. The only notable side effect was a slightly higher incidence of nausea in metformin-treated patients [89]. Importantly, multivariate analysis indicated that metformin use was an independent predictor of better response and lower relapse risk. This compelling evidence suggests that AMPK activation and metabolic interference can sensitize aggressive lymphoma to chemotherapy. It is hypothesized that metformin may inhibit lymphoma cell oxidative metabolism or mTOR signaling, as DLBCL cells often have high PI3K/mTOR activity, enhancing chemo-induced apoptosis. Based on these results, larger Phase 3 trials are being considered to confirm the benefit and potentially establish metformin as part of front-line DLBCL therapy [89].

In CLL, a more indolent lymphoma, metabolic interventions are being explored to prolong remission and overcome drug resistance. A Phase 2 pilot trial at the University of Michigan investigated metformin in relapsed/refractory CLL patients who are not yet on definitive therapy [87]. The rationale was that metformin might delay the need for cytotoxic treatment by suppressing CLL cell proliferation [87]. Clinically, a Spanish trial in CLL observed that diabetic patients on metformin had longer treatment-free survival. Metformin could serve as a low-cost maintenance therapy in CLL, keeping the disease in check by activating AMPK and forcing the leukemic cells out of their quiescent, protective metabolic state [87]. If successful, this could inaugurate a new strategy for managing early-stage CLL or enhancing the effect of emerging treatments like BTK inhibitors by tackling the leukemia’s metabolic adaptability.

### 3.7 AMPK-Targeted Novel Therapeutic Approaches

Across cancer types, an emerging theme is the combination of AMPK activators with immunotherapies. Metformin has been shown to modulate the tumor immune microenvironment by lowering immunosuppressive factors like IL-6 and VEGF and reprogramming T-cell metabolism toward memory/effector phenotypes [131]. Early clinical data in lung and liver cancers suggest that diabetic patients on metformin may respond better to PD-1 checkpoint inhibitors [132]. Building on these observations, new trials are being designed to formally test whether adding metformin can improve immunotherapy outcomes; for example, a study combining metformin with anti-PD1 in melanoma is under consideration. Moreover, other AMPK-activating agents, such as phenformin, are being evaluated in Phase 1 trials alongside immunotherapy, particularly in melanoma and NSCLC [133]. This strategy aims to leverage metabolic stress to make cancer cells more immunogenic or to relieve tumor-induced immunosuppression, offering an exciting avenue to potentially amplify the effectiveness of existing treatments.

Beyond pharmacologic agents, dietary interventions that activate AMPK are also being explored in oncology. For instance, a pilot trial in breast cancer examined short-term fasting around the time of chemotherapy to assess whether it could enhance treatment response [134]. Early small-scale studies have reported that fasting is safe and may reduce chemotherapy side effects, though larger trials are needed to determine its impact on tumor outcomes [135]. In glioblastoma and other difficult cancers, ketogenic diets have been proposed to stress tumor metabolism and activate AMPK in normal tissues, potentially slowing tumor growth [136,137]. While preclinical data are intriguing, these diets are difficult for patients to maintain and have yet to show clear clinical benefit, remaining a niche but noteworthy approach within the broader landscape of AMPK-focused cancer therapy.

## 4 Current Challenges and Therapeutic Limitations of AMPK-Targeted Strategies

A major challenge in leveraging AMPK for cancer therapy lies in its context-dependent, dualistic role-it can both suppress tumors and, in some cases, support their survival under stress. On one hand, AMPK activation inhibits cell growth and proliferation, functioning as a tumor suppressor [35]. The AMPK pathway is often downregulated or disrupted through loss of LKB1 in many cancers. On the other hand, tumor cells experiencing metabolic stress frequently rely on a baseline AMPK activity to adapt and survive. In such contexts, AMPK acts as a survival mechanism for cancer cells [24] (Fig. 2).

For example, in a KRAS-driven lung cancer model, treatment with phenformin significantly prolonged survival in mice-but only when the tumors lacked LKB1. Tumors with intact LKB1-AMPK signaling were more resistant to the metabolic stress induced by phenformin [35]. This suggests that cancer cells with a functional AMPK pathway may use it to endure hostile conditions like hypoxia or chemotherapy. These findings complicate AMPK-targeting therapies: while AMPK activation can initially inhibit tumor growth, in some cases, inhibiting AMPK may help eliminate highly stressed cancer cells. Therefore, AMPK activation is not always the clear therapeutic goal.

Current AMPK-targeting treatments face limitations in selectivity, bioavailability, and toxicity. Metformin, although widely used and safe for diabetes, is a relatively weak AMPK activator [138]. It requires high concentrations to significantly stimulate AMPK and works indirectly by inhibiting mitochondrial Complex I [139]. Moreover, tumor uptake of metformin is variable, and in non-diabetic cancer patients, its dosing is constrained by gastrointestinal side effects and the risk of lactic acidosis [140]. Phenformin, a more potent analog, activates AMPK more effectively through mitochondrial inhibition. However, it was withdrawn from clinical use in the 1970s due to cases of life-threatening lactic acidosis. This risk makes systemic phenformin use particularly dangerous, especially in patients with comorbidities [141].

Even metformin, while safer, can rarely cause lactic acidosis, especially with long-term use. Other AMPK activators also come with off-target effects. For instance, salicylate activates AMPK but also inhibits COX-1 and COX-2, which can lead to gastrointestinal bleeding at chemo-preventive doses [142]. The SGLT2 inhibitor canagliflozin activates AMPK indirectly by inducing glucose starvation, but its use may cause systemic effects such as ketoacidosis or weight loss [143]. A common issue with many of these compounds is a lack of specificity-they often activate AMPK as part of a broader metabolic disturbance rather than directly targeting the pathway.

This lack of selectivity can harm normal tissues: widespread AMPK activation may impair normal cell growth or lead to side effects such as fatigue and hypoglycemia [144]. Therefore, there is a critical need for more selective AMPK activators that specifically target tumor cells or the AMPK pathway without causing widespread metabolic disruption. Although some new allosteric AMPK activators and prodrugs are under investigation, challenges related to their bioavailability and efficacy have so far limited their clinical translation (Table 1).

## 5 Conclusion

AMPK plays a central role in maintaining cellular energy homeostasis, acting as a metabolic checkpoint that suppresses tumor growth by inhibiting anabolic pathways and promoting catabolic processes. Since cancer cells often depend on dysregulated metabolism to support rapid proliferation, AMPK activation has emerged as a promising strategy to counteract tumor progression. Both clinical and preclinical studies using activators such as metformin, phenformin, and canagliflozin have shown encouraging results in specific cancer types, including HER2-positive breast cancer, colorectal cancer, and certain metabolic subtypes of lung cancer.

However, their clinical utility of these agents remains limited by several factors. Many AMPK activators act indirectly and require high concentrations to achieve therapeutic effects, raising concerns about off-target actions and systemic toxicity. Additionally, tumor heterogeneity, differences in metabolic dependency, and the absence of robust biomarkers complicate patient stratification and treatment outcomes. While some cancers appear responsive to AMPK modulation, others may resist or adapt to energy stress, highlighting the need for better mechanistic insights.

To overcome these barriers, future research should prioritize combination therapies, precision targeting based on tumor metabolic profiles, and the development of more direct and selective AMPK modulators. mRNA-based approaches represent an innovative avenue for achieving controlled and tumor-specific AMPK activation, although challenges related to delivery and immunogenicity remain to be addressed. Ultimately, refining AMPK-targeted strategies may provide a powerful tool in metabolic cancer therapy, particularly when integrated into personalized treatment frameworks.

## Data Availability

Data sharing is not applicable to this article as no datasets were generated or analyzed during the current study.

## Abbreviations

4EBP1: Eukaryotic translation initiation factor 4E-binding protein 1
5-FU: 5-Fluorouracil
ABCB1: ATP-binding cassette sub-family B member 1
ACC: Acetyl-CoA carboxylase
AICAR: 5-Aminoimidazole-4-carboxamide ribonucleoside
ALL: Acute lymphoblastic leukemia
AML: Acute myeloid leukemia
AMPK: AMP-activated protein kinase
BMI: Body mass index
CAB39: Calcium-binding protein 39
CAB39L: Calcium-binding protein 39-like
CaMKK: Calcium/calmodulin-dependent protein kinase kinase 2
CLL: Chronic lymphocytic leukemia
COX-2: Cyclooxygenase-2
CPT1: Carnitine palmitoyltransferase 1
CRC: Colorectal cancer
DLBCL: Diffuse large B cell lymphoma
EGFR: Epidermal growth factor receptor
F-2,6-BP: Fructose-2,6,-bisphosphate
FDG: Fluorodeoxyglucose
GLUT4: Glucose transporter 4
HAS2: Hyaluronan synthase 2
HCC: Hepatocellular carcinoma
HMG-CoA: 3-hydroxy-3-methylglutaryl coenzyme A
HNSCC: Head and neck squamous cell carcinoma
LAR: Long-acting release
mTORC1: Mammalian target of rapamycin complex 1
NAFLD: Nonalcoholic fatty liver disease
NASH: Nonalcoholic steatohepatitis
NET: neuroendocrine tumor
NMIBC: Non-muscle-invasive bladder cancer
NSCLC: Non-small cell lung cancer
OS: Overall survival
PDAC: Pancreatic ductal adenocarcinoma
PDCD4: Programmed cell death protein 4
PET: Positron emission tomography
PFS: Progression-free survival
PPARγ: Peroxisome proliferator-activator gamma
PRKAB1: 5^′^-AMP-activated protein kinase subunit beta-1
PRKAB2: 5^′^-AMP-activated protein kinase subunit beta-2
PRKAA1: 5^′^-AMP-activated protein kinase catalytic subunit alpha-1
PRKAA2: 5^′^-AMP-activated protein kinase catalytic subunit alpha-2
PTEN: Phosphatase and tensin homolog
RCC: Renal cell carcinoma
RECIST: Response evaluation criteria in solid tumors
ROS: Reactive oxygen species
SGLT2: Sodium-glucose co-transporter 2
STK11: Serine/Thereonine Kinase 11
STRADA: STE20-related kinase adaptor alpha
STRADB: STE20-related kinase adaptor beta
TACE: Transarterial chemoembolization
TKI: Tyrosine kinase inhibitor
TME: Tumor microenvironment
TNBC: Triple-negative breast cancer
ULK1: Unc-51-like autophagy-activating kinase 1
WHO: World Health Organization
